# Total Wrist Arthroplasty for Posttraumatic Wrist Osteoarthritis: A Cohort Study Comparing Three Indications

**DOI:** 10.3390/life12050617

**Published:** 2022-04-21

**Authors:** Matthias Holzbauer, Julian A. Mihalic, Michael Pollak, Stefan M. Froschauer

**Affiliations:** 1 Department of Orthopedics and Traumatology, Kepler University Hospital, Krankenhausstrasse 9, 4020 Linz, Austria; matthias.holzbauer@a1.net (M.H.); julian.mihalic@kepleruniklinikum.at (J.A.M.); michael.pollak@kepleruniklinikum.at (M.P.); 2 Medical Faculty, Johannes Kepler University Linz, Altenberger Str. 69, 4040 Linz, Austria

**Keywords:** distal radius fracture, ReMotion prosthesis, scaphoid nonunion advanced collapse, scapholunate advanced collapse, total wrist arthroplasty, posttraumatic wrist osteoarthritis

## Abstract

Scapholunate ligament ruptures and scaphoid nonunion with consecutive advanced collapse (SLAC and SNAC wrists) as well as intra-articular distal radius fractures (DRF) are prone to cause posttraumatic wrist osteoarthritis. The aim of this study was to compare the outcomes of these indications for total wrist arthroplasty. We included 13, 11, and 8 patients with an overall mean age of 60 ± 9 years in the SLAC, SNAC, and DRF cohort, respectively. After an average follow-up period of 6 ± 3 years, we found no difference between our groups regarding pain levels and functional scores, although these parameters significantly improved compared to preoperative parameters. Complication and revision rates revealed no significant difference. However, significantly higher extension, arc of range of motion values in the flexion-extension, as well as in radial-ulnar deviation plain were detected in the SLAC compared to the DRF group. Finally, TWA proved to show a beneficial performance in all three investigated indications.

## 1. Introduction

Wrist osteoarthritis (OA) is a disabling condition that can be caused by a myriad of various etiologies. Systemic autoimmune-mediated conditions, e.g., rheumatoid arthritis, which leads to wrist OA in 90% of the cases within the first 10 years after its onset, is one main category [1]. Moreover, congenital wrist abnormalities, e.g., Madelung’s deformity, and vascular supply issues of the lunate and scaphoid causing Kienböck’s and Preiser’s disease, respectively, are considered as idiopathic causes [2]. Furthermore, wrist injury is another main reason for wrist OA [3]. The term posttraumatic wrist OA itself can be subdivided into the anatomical structure affected by the causative trauma. In this regard, a classification into ligament injuries and fractures is common, although Kienböck’s and Preiser’s disease are also discussed to be caused by a single impact or repetitive micro-trauma to the nutrient artery [3].

Weiss and Rodner summarized the generally accepted most common causes of posttraumatic wrist OA in three categories: scapholunate advanced collapse (SLAC), scaphoid fracture nonunion advanced collapse (SNAC), and OA secondary to an intra-articular fracture of the distal radius or ulna, or from an extra-articular fracture resulting in malunion and abnormal joint loading [2].

Historically, total wrist arthroplasty (TWA) has been introduced for patients suffering from rheumatoid arthritis [4]. A limited number of studies are available focusing solely on non-rheumatoid study populations [5,6,7,8,9,10,11]. Studies involving only posttraumatic cases are even more scarce [7,9,10,11]. In particular, the literature lacks evidence of if the kind of underlying trauma causing OA has an impact on the performance of TWA. Thus, the null hypothesis of the present study was that there is no difference in the main outcome parameter, i.e., DASH scores, between SLAC, SNAC, and DRF patients treated with ReMotion TWA for posttraumatic wrist OA.

Therefore, we aimed to gain further insights in proper indications for total wrist prosthesis implantation.

## 2. Materials and Methods

**Figure 1:**
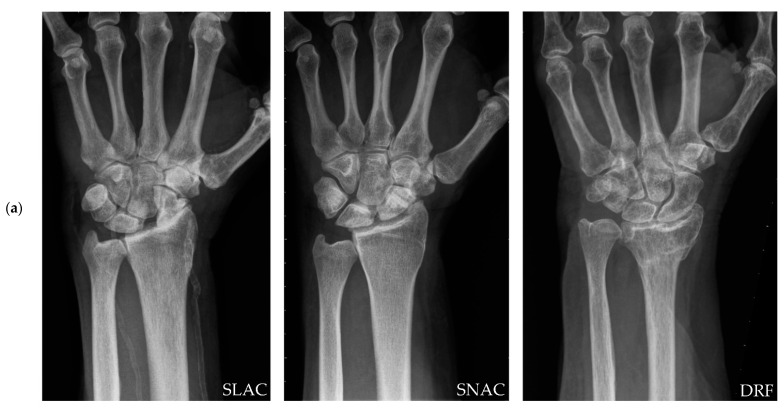

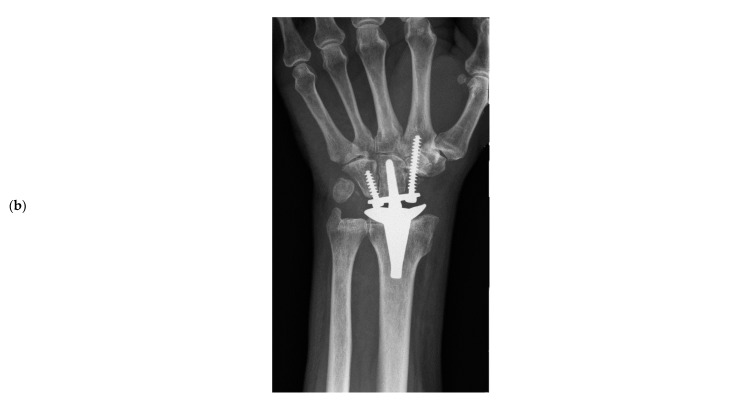
(**a**) Preoperative X-rays of one patient each of our three study cohorts (the indications are noted at bottom right corner of the images) (**b**) Postoperative X-ray 1 month after ReMotion TWA using the PRC technique.

All patients who underwent TWA between July 2007 and October 2019 were retrospectively reviewed from our database. At our institution, stage three OA according to the Kellgren–Lawrence classification was a prerequisite for total wrist joint replacement [12]. Posttraumatic indications for TWA were included in the present study, i.e., SLAC wrists, SNAC wrists, and DRFs leading to wrist OA (Figure 1a). Only patients with a minimum postoperative follow-up period of more than 1 year were included. Moreover, pre- and postoperative clinical as well as radiographic parameters (see Section 2.1) had to be available.

Limited wrist arthrodesis or proximal row carpectomy (PRC) surgery before TWA were defined as exclusion criteria. However, SLAC patients who had received scapholunate ligament reconstruction, SNAC patients with previous Herbert screw fixation, and DRF patients with volar locking plate implantation were eligible to participate in this study. Moreover, cases with wrist denervation surgery were not excluded from this investigation. The number of previous surgeries aiming to treat the underlying traumatic lesion were noted.

All patients received TWA using the ReMotion Total Wrist System (Stryker, Kalamazoo, Michigan). Several cases were already included in our previous study reporting non-rheumatoid patients [6] as well as the study comparing the PRC technique to the conventional carpal resection method [13]. One level IV surgeon performed the former (Figure 1b) and two other level IV surgeons performed the latter technique [14].

The study was approved by the Institutional Review Board (Ethics Commission of Johannes Kepler University Linz #1082/2021) and conducted in accordance with the guidelines of the Declaration of Helsinki. Written informed consent was obtained from all subjects involved in the study.

### 2.1. Clinical and Radiographic Evaluation

Preoperative assessment involved active range of motion (ROM) measurements via flexion, extension, radial, and ulnar deviation. Furthermore, all patients filled in the disabilities of arm, shoulder, and hand (DASH) questionnaire. Pain was evaluated using a visual analogue scale (VAS) ranging from 0 (no pain) to 10 (worst imaginable pain).

At the final follow-up examination, these assessments were repeated. Additionally, bilateral grip strength measurement was performed. Moreover, patient satisfaction was evaluated using the question of whether the patients would undergo surgery again.

All occurring clinical complications at any follow-up time were recorded. A final complication rate was calculated for each cohort. Revision surgery was considered as any change of components or explantation, while reoperations were defined as any other surgery treating a complication related to TWA implantation.

Intraoperative as well as final radiographs were assessed according to the method introduced by Boeckstyns et al. [15]. Thus, the angle between the radial component’s stem and the long axis of the radius (*Implant-Radius angle*) as well as the angle between the carpal peg and the axis of the third metacarpal (*Implant-MCIII angle*) were measured. Moreover, the distance between the tip of the radial implant and the tip of the radial styloid (*Implant-Styloid distance*) as well as the distance between the tip of the carpal peg and the base of the third metacarpal (*Implant-BasisMCIII distance*) were evaluated.

Final radiographs were additionally screened for screw breakage. Furthermore, radiolucency adjacent to the radius as well as the carpal implant, i.e., signs of reduced bone mass compared to the intraoperative radiograph, were recorded.

### 2.2. Statistical Methods

The arc of ROM was calculated by adding up flexion and extension angles as well as radial and ulnar deviation angles, respectively. The *difference in grip strength* represents the subtraction of the grip strength of the operated hand from the healthy hand.

The Kolmogorov-Smirnov test was performed to test the normality of all outcome parameters within each cohort. In case of normal data, values were presented as mean ± standard deviation, while skewed data were displayed as median (interquartile range).

Comparative testing between the three cohorts was performed using the one-way analysis of variances (ANOVA) for normal data and the Kruskal-Wallis test for skewed data. If these tests revealed a significant result, pairwise post hoc tests were conducted and the adjusted significance values according to the Dunn-Bonferroni correction were reported. Dichotomous and nominal variables were tested using the chi-squared test or the Fisher′s exact test if any value was <5.

Pre- and postoperative values within each cohort were compared using the dependent Student’s *t*-test for normal data and Wilcoxon test for skewed data.

A *p*-value smaller than 0.05 was considered as significant.

Moreover, according to the main outcome parameter, i.e., DASH scores, we performed a post hoc power analysis using GPower.

## 3. Results

Our database review revealed that 36 patients were treated with ReMotion TWA for the three indications included in the present study. However, four patients had to be excluded because they received the following surgeries before TWA implantation: four-corner fusion (*n* = 2), scapholunate fusion (*n* = 1), and PRC (*n* = 1). Thus, 32 patients whose demographics are displayed in Table 1 could be analyzed in the present study. Details regarding the surgical technique, the implanted prosthesis size, follow-up years, as well as the number of previous surgeries are presented. We also screened the medical records for the time between the wrist trauma and total joint replacement, while not all patients could remember the causative injury.

Statistical testing revealed that patient demographics were homogenously distributed.

Clinical and radiographic outcome parameters are summarized in Table 2. Furthermore, *p*-values resulting from statistical analyses were reported. We fund that there was no statistical difference in the preoperative values between the three cohorts. Regarding postoperative comparisons, the significant parameters were further analyzed in pairwise comparisons: we found significantly lower arc of ROM (Felx. + Ext.) (*p* = 0.004) and lower arc of ROM (Rad. + Uln. Dev.) (*p* = 0.03) values in the DRF cohort compared to the SLAC group. Regarding extension, pairwise comparisons showed significantly decreased values in the SNAC (*p* = 0.03) and DRF (*p* = 0.001) cohort compared to the SLAC cohort.

Table 3 displays a longitudinal statistical work-up of clinical outcome parameters.

Regarding complications, we detected two radial impaction syndromes in the SLAC cohort. In the SNAC cohort, we recorded complications in six patients leading to one revision surgery and two reoperations: a loosening of the carpal implant resulted in a conversion to an arthrodesis in one patient. Moreover, an early postoperative wound infection caused the necessity of a wound revision including free flap covering. An ulnar impingement syndrome was treated with a Darrach procedure. In another patient, radial impaction syndrome and asymptomatic radial screw breakage were detected. Furthermore, one patient suffered from radial impaction syndrome, and one presented with De Quervain′s tenosynovitis. In the DRF cohort, we recorded four complications: one patient required radial screw change after symptomatic screw breakage. Three patients suffered from radial impaction syndrome, while one patient received scaphoidectomy and one received de Quervain’s tendon release due to accompanying tendosynovitis. All complications not requiring revision surgery or reoperation could be resolved using conservative treatment.

Radiographically, we detected three asymptomatic breakages of the radial screw in the SLAC cohort.

Post hoc power analysis based on the previously displayed DASH scores revealed a value of 0.89.

## 4. Discussion

In the present study, the null hypothesis was accepted that the three included indications (SLAC, SNAC, and DRF) for ReMotion implantation resulted in no significant difference regarding postoperative functional scores. Generally, outcome parameters showed similar values in the three groups. Functional impairment and pain levels could be substantially improved in all cohorts. Regarding ROM data, reduced values in terms of extension as well as both arc of ROM measurements could be detected in the DRF group. We suspect that the underlying trauma leading to DRFs, which we encounter for the most common types of fracture [16], has a higher impact on the ligamentous integrity and biomechanical function compared to trauma causing scapholunate ligament ruptures and scaphoid fractures. The subsequent cast immobilization or immobilization after open reduction and internal fixation might additionally cause increased capsuloligamentous scarring, adhesions, and consecutive ROM restrictions. Moreover, Cottias et al. reported that DRFs with an intra-articular step of more than 2 mm are prone to cartilage lesions and consecutive development of wrist OA [17]. Therefore, intraarticular DRFs imply an increased difficulty of anatomical reconstruction in acute trauma treatment as well as a higher risk for the development of subsequent wrist OA. This risk increases with the grade of comminution or a higher rating according to the Arbeitsgemeinschaft für Osteosynthesefragen/Orthopaedic Trauma Association (AO/OTA) system [18]. Although wrist hemiarthroplasty is also available for acute treatment of DRFs, the indication for this procedure is currently reserved for severely comminuted and irreparable fractures [19,20]. Initial treatment using volar fixed-angle plate systems and cast immobilization showed an equally good short-term performance [21]. Therefore, TWA might serve as salvage treatment in the follow-up course if active patients face ongoing problems during their everyday life.

After non-surgical treatment options have been exhausted, various treatment approaches for posttraumatic wrist OA aim to both achieve pain relief and preserve as much motion as possible at an acceptable complication rate [2,22]. Historically, pancarpal degenerative wrist OA was generously treated with total wrist arthrodesis, especially in patients still wishing to perform heavy labor [23]. This method’s main disadvantage is immobility leading to limited functional outcome scores [24]. Thus, a wide range of limited wrist arthrodesis techniques with specially designed osteosynthesis material were developed. Scaphotrapezium-trapezoid arthrodesis, scaphocapitate arthrodesis, radioscapholunate arthrodesis, scapholunocapitate arthrodesis, and four-corner (capitate–lunate–hamate–triquetrum) arthrodesis, for instance, involve a more differentiated approach; hence, fusion is only performed in arthritically degenerated wrist joints while motion is maintained in a portion of the wrist which is not affected by OA itself [2,25,26]. In this regard, the sequence of chondral destruction caused by SLAC and SNAC wrist has been previously described in four stages: these areas are generally quite similar within these two degenerative wrist diseases [26]. While four-corner arthrodesis is a widespread surgery used for these two indications, proximal row carpectomy renders another more cost-effective and less surgically demanding surgical technique [26,27]. However, predicted loss of motion and subsequent OA of the more stressed joints are well-known drawbacks of these two surgical methods [24,26].

As a result, total wrist arthroplasty (TWA) has become an increasingly attractive procedure to achieve the intended goals of pain relief and preserved ROM [2,22,24]. Physiological kinematics of the global wrist motion is a complex topic where several models and theories simultaneously exist, although advanced biomechanical analysis techniques are currently used [28]. Therefore, it seems obvious that detailed biomechanical reconstruction cannot be accomplished using prosthetic joint replacement. In contrast to the previously presented surgical alternatives, however, TWA using advanced implant designs with ellipsoidal design, e.g., ReMotion prosthesis, can reconstruct physiological motion as best as possible. In particular, one can even find similarities between these prostheses’ function and the Ovoid-/C Shape wrist motion theory [29,30].

Furthermore, TWA is independent from the definite area of chondral wrist destruction, which plays a pivotal role in limited wrist arthrodeses. In this regard, we advocated for the PRC implantation technique, especially in SLAC and SNAC wrists. Thereby, radial impaction syndrome can be significantly reduced [13] and issues related to the sclerotic deformed scaphoid can be prevented due to its total removal. Recent advances in TWA’s implant design and materials have massively improved the initially high complication rates [24]. This can be seen while comparing our outcomes to previous studies conducted on TWA for solely posttraumatic indications: the Biax prosthesis, i.e., a second-generation implant, showed a high dislocation and consecutive revision rate [8]. Finally, the last-generation Destot prosthesis, which is no longer available, presented even better ROM values compared to the present study [9]. Moreover, Reigstad et al. and Boeckstyns et al. reported their results for TWA using the Motec and ReMotion implant, respectively: their outcomes corroborate our findings, that favorable ROM as well as pain relief and functional improvement can be achieved using TWA for posttraumatic indications [10,11]. Moreover, our overall revision surgery rate of 6% as well as the rate within every cohort is in the range of the previously mentioned two studies (20% and 4%).

The present study involved several limitations. Although the three cohorts showed homogenously distributed demographics as well as preoperative data, the number of included patients for each indication was relatively low. However, the sample size of the included cohorts resulted in an acceptable post hoc power value. Due to the retrospective character of the study, the patients had a different history of previous therapy or surgery within every cohort. Due to a lack of data, we could not provide a grading of the carpal instability, e.g., using the Mayfield classification [31]. However, the radiographic stage and location of OA was a common prerequisite for all prosthesis implantations at our institution. The radiographic measures implied a certain degree of inaccuracy because the long axis of the radius and the MC III could not be referenced to exact osseous landmarks and the *Implant-Styloid Distance* was influenced by the growth of osteophytes at the radial styloid. Moreover, the clinical relevance of periprosthetic radiolucency is still not fully understood. Furthermore, the simple presence of reduced bone mass is hard to quantify using plain radiographs.

In conclusion, we accepted our null hypothesis: our outcomes showed that DRSs, SLAC, and SNAC wrists causing posttraumatic OA revealed no difference in postoperative performance after ReMotion implantation. The aimed purpose of the procedure, i.e., pain relief, functional improvement, as well as preserving motion, could be achieved in all three cohorts. Thus, we recommend TWA for posttraumatic OA in the three investigated indications as a beneficial procedure.

## Figures and Tables

**Table 1:** Patient demographics.

Parameters	SLAC	SNAC	DRF	*p*-Value
Patients	13	11	8	-
Wrists	13	11	8	-
Age	63.4 ± 8.0	56.7 ± 9.9	57.5 ± 8.8	0.16 ^1^
Sex (f/m)	3/10	3/8	6/2	1.00 ^2^
Side (l/r)	2/11	5/6	3/5	0.24 ^2^
Technique (CCR/PRC)	6/7	6/5	6/2	0.50 ^2^
Prosthesis size (S/M/L)	4/6/3	4/7/0	1/7/0	-
Inlay (N/Ext)	11/2	10/1	6/2	-
Screw II MC	18 (4)	18 (4)	18 (3)	-
Screw IV MC	30 (0)	30 (0)	30 (0)	-
Follow-up (years)	4.5 ± 2.9	6.8 ± 3.3	7.4 ± 3.0	0.09 ^3^
Previous surgery	0 (1)	1 (1)	1 (1)	0.25 ^3^
Trauma (years)	6.8 (8.7)	15.6 ± 13.7	1.7 (4.1)	0.12 ^3^
	*n* = 4	*n* = 10	*n* = 8	

Statistical testing was performed using ^1^ one-way ANOVA, ^2^ Fisher’s exact test, and ^3^ Kruskal-Wallis test.

**Table 2:** Outcome parameters.

Parameters	Time	SLAC	SNAC	DRF	*p*-Value
		*n* = 13	*n* = 11	*n* = 8	
DASH scores	preop.	62 ± 11	60 ± 15	69 ± 16	0.33 ^1^
	postop.	24 (36)	31 ± 26	35 ± 21	0.57 ^2^
VAS for pain	preop.	7 (1)	7.0 ± 1.5	6.9 ± 1.1	0.86 ^2^
	postop.	2.2 ± 1.9	3.1 ± 2.4	3.6 ± 1.9	0.33 ^1^
Flexion	preop.	30 (15)	30 (10)	19 ± 12	0.14 ^2^
	postop.	39 ± 11	35 (10)	31 ± 9	0.24 ^2^
Extension	preop.	20 (15)	23 ± 7	20 ± 8	0.72 ^2^
	postop.	38 ± 7	30 (5)	24 ± 9	0.001 ^2^
Arc of ROM (Flex. + Ext.)	preop.	52 ± 16	50 ± 14	39 ± 18	0.23 ^1^
	postop.	77 ± 14	63 ± 12	56 ± 15	0.004 ^1^
Radial deviation	preop.	10 (5)	7 ± 6	8 (5)	0.42 ^2^
	postop.	14 ± 5	15 ± 7	10 (8)	0.40 ^2^
Ulnar deviation	preop.	15 (5)	15 (5)	13 (5)	0.16 ^2^
	postop.	26 ± 5	25 ± 8	18 ± 9	0.06 ^1^
Arc of ROM (Rad. + Uln. Dev.)	preop.	27 ± 9	21 ± 7	21 ± 6	0.11 ^1^
	postop.	40 ± 8	35 (10)	29 ± 9	0.03 ^2^
Grip strength operated hand	postop.	30 ± 13	26 ± 12	23 ± 12	0.43 ^1^
Grip strength healthy hand	postop.	39 ± 17	37 ± 18	44 (14)	0.91 ^2^
Difference in grip strength	postop.	8 ± 11	11 ± 9	16 ± 8	0.18 ^1^
Satisfied	postop.	13 (100%)	10 (91%)	7 (88%)	0.50 ^3^
Complication	postop.	2 (15%)	6 (54%)	4 (50%)	0.11 ^3^
Reoperation	postop.	0 (0%)	2 (18%)	2 (25%)	0.15 ^3^
Revision	postop.	0 (0%)	1 (9%)	1 (13%)	0.50 ^3^
Implant-Radius angle	intraop.	7 ± 5	6 ± 3	7 ± 3	0.72 ^1^
	postop.	7 ± 5	6 ± 4	7 ± 2	0.68 ^1^
Implant-MCIII angle	intraop.	7 ± 4	6 ± 5	6 ± 3	0.82 ^1^
	postop.	6 ± 3	8 ± 6	7 ± 4	0.51 ^1^
Implant-Styloid distance	intraop.	39 ± 4	39 ± 3	37 ± 4	0.29 ^1^
	postop.	41 ± 4	40 ± 3	38 ± 5	0.17 ^1^
Implant-BasisMCIII distance	intraop.	5 (2)	4 ± 3	3 ± 3	0.29 ^1^
	postop.	4 (3)	3 ± 3	2 ± 3	0.65 ^2^
Radial radiolucency	postop.	5 (38%)	6 (54%)	5 (63%)	0.48 ^3^
Carpal radiolucency	postop.	6 (46%)	4 (36%)	2 (25%)	0.66 ^3^

Statistical testing was performed using ^1^ one-way ANOVA, ^2^ Kruskal-Wallis test, ^3^ Fisher’s exact test; preop. = preoperative; postop. = postoperative.

**Table 3:** *p*-values resulting from the comparison between pre- and postoperative outcomes within every group.

Parameters	SLAC	SNAC	DRF
DASH scores	0.001 ^2^	<0.0001 ^1^	<0.0001 ^1^
VAS for pain	0.001 ^2^	<0.0001 ^1^	0.003 ^1^
Flexion	0.02 ^2^	0.14 ^2^	0.04 ^1^
Extension	0.002 ^2^	0.03 ^2^	0.13 ^1^
Arc of ROM (Flex. + Ext.)	0.001 ^1^	0.02 ^1^	0.03 ^1^
Radial deviation	0.02 ^2^	0.007 ^1^	0.10 ^2^
Ulnar deviation	0.008 ^2^	0.009 ^2^	0.16 ^2^
Arc of ROM (Rad. + Uln. Dev.)	0.0003 ^1^	0.005 ^2^	0.03 ^1^
Implant-Radius angle	0.27 ^1^	0.44 ^1^	0.69 ^1^
Implant-MCIII angle	0.15 ^1^	0.19 ^1^	0.32 ^1^
Implant-Styloid distance	0.17 ^1^	0.20 ^1^	0.46 ^1^
Implant-Basis-MCIII distance	0.01 ^2^	0.14 ^1^	0.25 ^1^

Statistical testing was performed using ^1^ paired Student’s *t*-test and ^2^ Wilcoxon test.

## Data Availability

The data that support the findings of this study are available from the first author (S.F.), upon reasonable request.
